# Viral and cellular translation during SARS‐CoV‐2 infection

**DOI:** 10.1002/2211-5463.13413

**Published:** 2022-04-25

**Authors:** Gilbert Eriani, Franck Martin

**Affiliations:** ^1^ Architecture et Réactivité de l’ARN CNRS UPR9002 Institut de Biologie Moléculaire et Cellulaire Université de Strasbourg France

**Keywords:** immune response, interferon, NSP1‐SL1, ribosome, SARS‐CoV‐2, translation

## Abstract

SARS‐CoV‐2 is a betacoronavirus that emerged in China in December 2019 and which is the causative agent of the Covid‐19 pandemic. This enveloped virus contains a large positive‐sense single‐stranded RNA genome. In this review, we summarize the current knowledge on the molecular mechanisms for the translation of both viral transcripts and cellular messenger RNAs. Non‐structural proteins are encoded by the genomic RNA and are produced in the early steps of infection. In contrast, the structural proteins are produced from subgenomic RNAs that are translated in the late phase of the infectious program. Non‐structural protein 1 (NSP1) is a key molecule that regulates both viral and cellular translation. In addition, NSP1 interferes with multiple steps of the interferon I pathway and thereby blocks host antiviral responses. Therefore, NSP1 is a drug target of choice for the development of antiviral therapies.

## Graphical abstract

This Review gives an overview of translation during SARS‐CoV‐2 infection. This encompasses molecular mechanisms for viral translation of both genomic and subgenomic transcripts. Host translation is shut down by NSP1‐mediated mechanisms. mRNAs involved in type I interferon antiviral response are specifically silenced. Different functions of NSP1 as an essential virulent factor are discussed for the main coronavirus families.

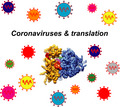

**Abbreviations**
Covid‐19: coronavirus disease 2019
CrPV: cricket paralysis virus
Cryo‐EM: cryo‐electronic microscopy
dsRNA: double‐stranded RNA
eIF: eukaryotic Initiation Factor
IFN: interferon
ISG: IFN‐stimulated gene
ISRE: IFN‐I‐stimulated response element
MERS‐CoV: middle east respiratory syndrome coronavirus
mRNA: messenger RNA
NSP: non‐structural proteins
PFSE: programmed −1 frameshift stimulation element
SARS‐CoV‐2: severe acute respiratory syndrome coronavirus 2
TRS: transcription Regulatory Sequence
uORF: upstream Open Reading Frame
UTR: unstranslated Region

In the kingdom *Orthornavirae*, the order of *Nidovirales* comprises the Coronaviruses that belong to the *coronaviridae* family. These viruses are a serious threat to public health all over the world. Coronaviruses are among the largest RNA viruses. Their positive‐sense single‐stranded genomic RNAs are usually very long. Four genera have been described in the coronaviridae family: the *alpha*, *beta*, *gamma,* and *deltacoronaviruses*. Among the betacoronaviruses, five subgenera have been characterized so far: the *Embecovirus*, the *Hibecovirus*, the *Nobecovirus*, the *Merbecovirus,* and the *Sarbecovirus* [1, 2, 3]. Recently, highly pathogenic human coronaviruses from the betacoronavirus genus have caused serious epidemic outbursts in the last few decades. First, the Severe Acute Respiratory Syndrome coronavirus SARS‐CoV (nowadays called SARS‐CoV‐1) emerged in southern China in 2002 and caused a world epidemic in 2003 [4, 5]. Then, in 2012, the Middle East respiratory syndrome coronavirus (MERS‐CoV), which belongs to the *Merbecovirus* subgenus, was first identified in Saudi Arabia and was the causative agent of the so‐called viral respiratory disease MERS [6, 7]. And recently, in December 2019, the SARS‐CoV‐2 emerged in Wuhan in China and led to the Covid‐19 pandemic [8, 9]. SARS‐CoV‐1 and SARS‐CoV‐2 are both members of the same subgenus *Sarbecovirus* from the betacoronaviruses family. Early reports mentioned that genomic positive‐sense RNAs from the Sarbecovirus are large (26.2 to 31.7 kilobases), capped at their 5′ end and polyadenylated at their 3′ end [10, 11].

In the cell, the protein synthesis process takes place on the macromolecular machinery named the ribosome. The human ribosome is composed of the small 40S ribosomal subunit, which is the decoding site, and the large 60S ribosomal subunit, which contains the peptidyl transferase center that catalyzes peptide bond formation between amino acids of the nascent protein [12]. In the cell, canonical translation is a highly regulated process that can be subdivided into four steps: initiation, elongation, termination, and recycling [13]. First, translation initiation consists of the assembly of a complete ribosome 80S by joining the 40S and 60S subunits on the start codon. Then, the second step is elongation, during which the encoded peptide is assembled until termination occurs when the elongating ribosome meets the stop codon. After termination, the ribosomal subunits disassemble from the mRNA and undergo a so‐called recycling step to prepare the two ribosomal subunits for the next round of translation. Translation initiation is the rate‐limiting step; the precise localization of the AUG start codon is a critical event that requires numerous *trans*‐acting factors called eukaryotic Initiation Factors (eIFs) [13, 14].

As coronaviruses carry a positive‐sense genomic RNA, mRNA translation takes place directly on the viral genomic RNA molecule that is introduced into the cell during infection. Therefore, efficient viral translation by the host ribosomes is a critical early event for viral propagation. Concomitantly, host cellular translation is shut down to ensure that the translation machinery is hijacked and therefore exclusively dedicated to viral component synthesis. In this review, we will focus on the SARS‐CoV‐2 virus. We will summarize the current knowledge on translation of both viral transcripts and host cellular messenger RNAs during infection by SARS‐CoV‐2. We will also discuss the impact of SARS‐CoV‐2 virus entry on host antiviral defenses. The last section will be dedicated to comparisons of SARS‐CoV‐2 and other coronaviruses.

## Translation of viral transcripts

**Fig. 1:**
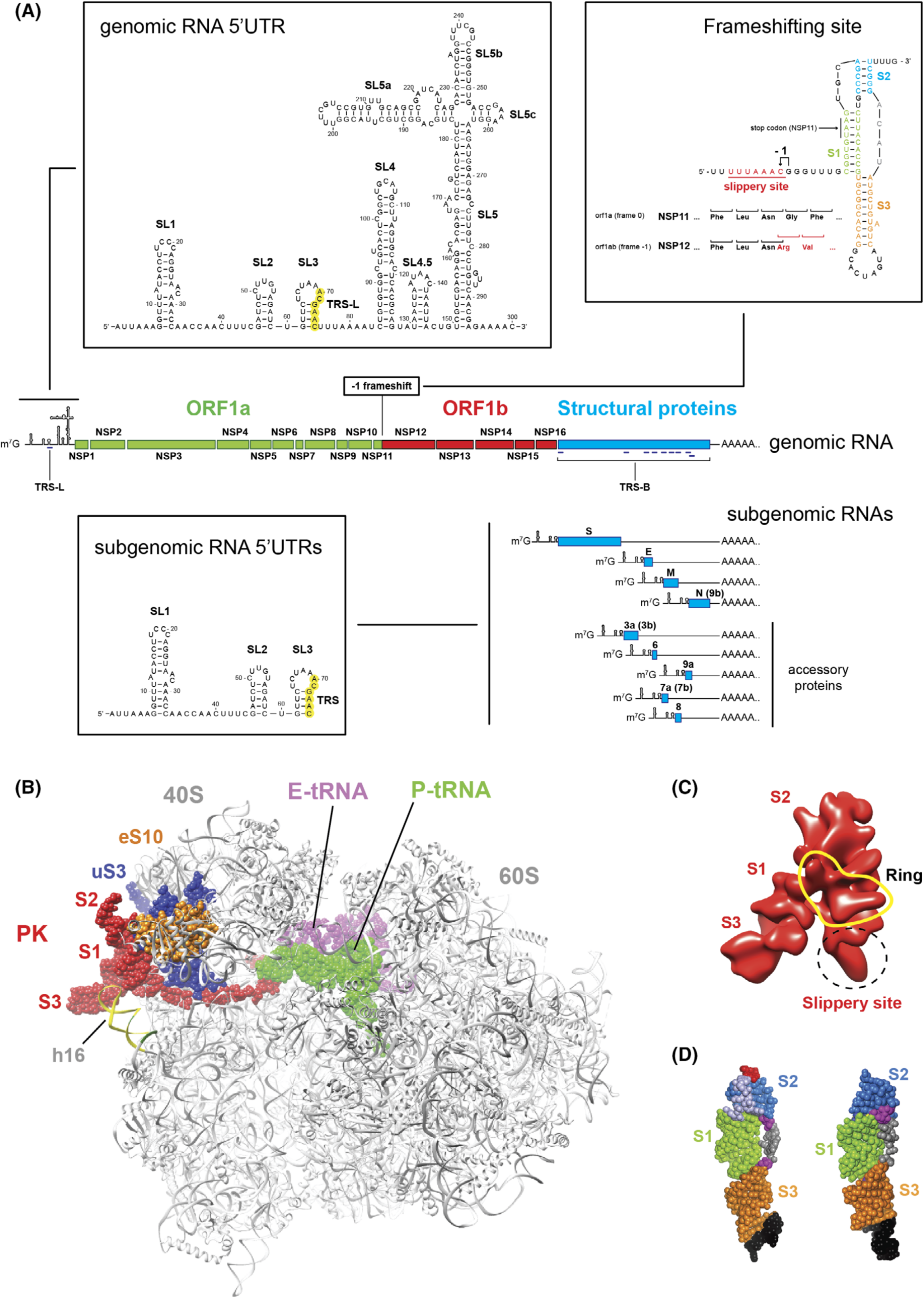
Cis‐acting elements on viral SARS‐CoV‐2 transcripts. (A) Genomic and subgenomic RNA transcripts. The secondary structures of the 5’UTRs of the genomic RNA and the subgenomic RNAs and the Programmed −1 Frameshift Stimulation Element (PFSE) are shown in boxes. The nucleotides of TRS‐L and TRS are shown in green in the 5’UTRs. In the PFSE, the pseudoknot consists of stems S1 (green), S2 (blue), and S3 (orange). The slippery site is shown in red and underlined. The −1 frameshifting site is indicated by a black arrow. The codons of NSP11 (frame 0) and NSP12 (frame −1) are shown under the nucleotide sequence. The NSP11 stop codon in S1 is indicated by a black arrow. In the subgenomic transcripts, proteins encoded by leaky scanning are indicated in brackets (B) Structure of a translating ribosome that pauses at the PFSE (PDB:7o7z) [24]. The 80S ribosome is shown gray. The PFSE and the slippery sequence are shown in red: It interacts with ribosomal proteins eS10 (orange) and uS3 (dark blue) and the 18S rRNA helix h16 (yellow). The E‐site tRNA is shown in pink and the P‐site tRNA is shown in green. (C) The cryo‐EM structure of the free PFSE is shown in red (EMD‐22296) [25]. The positions of stems S1, S2, and S3 are indicated. The slippery site is circled by a dashed line. The presence of a central ring is shown in yellow. (D) Crystallographic structures of the free PFSE (PDB:7mlx) [26] (left) and (PDB:7mky) [27] (right). The stems S1 (green), S2 (blue) and S3 (orange) are shown.

SARS‐CoV‐2 is an enveloped positive‐stranded RNA virus, and the assembled particles are in general 60 to 140 nm in diameter. The virus particles contain a large genomic RNA that is 29903 nucleotides long [8]. Like other coronaviruses, the genomic RNA is believed to be capped at the 5′ end and polyadenylated at the 3′ end [10, 11], although the presence of a canonical cap has not yet been demonstrated for the SARS‐CoV‐2 genome. The genome is divided in two parts: two‐thirds of the genome on the 5′ part codes for non‐structural proteins and one‐third of the genome on the 3′ part codes for structural proteins (Fig. 1A). The coding sequence for non‐structural proteins contains two large open reading frames, Orf1a and Orf1ab, which are both translated into two polyproteins. Orf1a enables the synthesis of a polyprotein that is further processed by proteolytic cleavages into non‐structural proteins NSP1 to NSP11. Translation of the second Orf1ab requires a −1 frameshifting event; the synthesized polyprotein is also processed by proteolytic cleavages to generate four additional non‐structural proteins, NSP12 to NSP16. NSP12 is the viral RNA‐dependent RNA polymerase, also called RdRp, which is required to synthesize the genomic and subgenomic RNA transcripts. NSP12 to NSP16 are involved in core enzymatic functions, such as synthesis, capping, modifying, and processing of viral transcripts. The secondary structures of the 5′ leader of the SARS‐CoV‐2 genome have been predicted [15] and experimentally determined by in solution probing [16] (Fig. 1A). The ~300 nucleotide long 5′ leader contains five stem loop structures named SL1 to SL5. SL5 is a large structure that contains a four‐way helix junction that encompasses the three hairpins SL5a, SL5b, and SL5c. Later on, the secondary structure model of the genomic RNA 5′ leader was also confirmed *in vivo* in cells infected by SARS‐CoV‐2 [17]. In the late phase of the infectious process, subgenomic RNAs are synthesized. Among the proteins encoded by the nine subgenomic RNAs, there are the structural proteins Spike (S), the Envelope (E), the Membrane (M), and the Nucleocapsid (N). Other subgenomic RNAs code for accessory proteins called orf3a, orf3b, orf6, orf7a, orf7b, orf8, and orf9b. Orf3b, orf7b, and orf9b are produced by leaky scanning of the sgRNAs coding for orf3a, orf7a, and nucleocapsid N, respectively. The subgenomic RNAs coding for N, S, orf7a, and orf3a are the most abundant [18]. The median length of the polyA tail of the subgenomic RNAs is 47 A residues; however, two populations in subgenomic RNAs can be distinguished with an average of 30 and 45 A residues, respectively [18]. Subgenomic RNAs are synthesized by the viral RNA‐dependent polymerase RdRp (NSP12), which uses Transcription Regulatory Sequences (TRSs). The TRSs located at the 5′ end of each subgenomic coding sequence are called TRS in the body (TRS‐B), and the unique TRS located in the 5′ leader is the TRS in the leader (TRS‐L) (Fig. 1A). When the RNA polymerase RdRp undergoes negative‐strand synthesis from the 3′ end of the genomic RNA, it pauses on TRS‐B sequences and switches the template to the TRS‐L by discontinuous transcription [19]. This unique mechanism leads to the fusion of the TRS‐B and TRS‐L sequences and allows the synthesis of negative‐strand templates that are used later on for the synthesis of positive‐stranded subgenomic RNAs. The consequence of this synthesis mechanism is that all the viral subgenomic RNAs share the same 5′ leader, which contains SL1, SL2, and SL3 (Fig. 1A).

The secondary structure of the site of −1 frameshifting in between NSP11 and NSP12 coding sequence has also been investigated thoroughly [20, 21, 22, 23]. The −1 frame shifting occurs on a Programmed −1 Frameshift Stimulation Element (PFSE) that exists in a so‐called slippery sequence located seven nucleotides upstream of a complex pseudo knot structure formed by three stems S1, S2, and S3 (Fig. 1A). The −1 frameshift allows the ribosome to avoid the NSP11 stop codon and therefore enables translation of the NSP12 coding sequence from orf1ab. Structural data obtained by Cryo‐EM and by X‐ray crystallography have shed light on mechanistic details of this frameshifting mechanism. The structure of a translating ribosome on the SARS‐CoV‐2 −1 frameshifting region obtained by Cryo‐EM revealed that the pseudo knot structure is located at the mRNA entry channel and interacts with ribosomal proteins uS3 and eS10, and the helix h16 of the 18S rRNA (Fig. 1B). This set of interactions induces tensions in the mRNA that are critical to promote −1 frameshifting [24]. In addition, the nascent polyprotein also interacts with ribosomal components in the peptide exit tunnel that further contribute to the frameshifting mechanism [24]. The structure of the whole PFSE alone has also been determined by cryo‐EM (Fig. 1C) and revealed its overall topology before the arrival of the translating ribosome. The structure contains a ring which allowed the design of antisense oligonucleotides that prevents −1 frameshifting, and thereby interferes with viral propagation [25]. Then, X‐ray crystallography studies showed that the structure of the pseudoknot is formed by three H‐type stems stacked in a vertical orientation (Fig. 1D): These structures bring interaction details at atomic resolution that will be useful for the identification of binding sites of specific ligands and for the drug design of antiviral compounds that will target specifically the PFSE [26, 27]. In addition, a short isoform of the host zinc‐finger antiviral protein ZAP‐S directly interacts with the PFSE and thereby modifies its folding, leading to downregulation of −1 frameshifting [28]. The genomic and subgenomic RNAs present in the host cell during SARS‐CoV‐2 infection are translated by the human host ribosomes. Translation of viral transcripts has been assessed by ribosome profiling approaches [29]. As already mentioned, non‐structural proteins are exclusively produced by translation of the genomic RNA. Several distinct methods have enabled evaluation of the frameshifting rate in coronaviruses to be between 25 and 75% in coronaviruses [21, 22, 30, 31]. In the case of SARS‐CoV‐2, the method consists of dividing the ribosome footprint density of orf1ab in the −1 frame by the density observed in orf1a in the 0 frame; the −1 frameshifting rate led to the estimation of around 57% frameshifting [32]. This value is comparable to the frameshifting rate observed in other viruses such as Mouse Hepatitis Virus (MHV) [31]. Such a high frameshifting rate indicates that the frameshifting is very efficient and fast, and therefore, frameshifting does not lead to ribosome arrest. As for Infectious Bonchitis Virus (IBV), no ribosomal pause at the frameshifting site was observed in the infection of SARS‐CoV‐2, thereby corroborating the high frameshifting rate [33]. Interestingly, the stoichiometry of subgenomic RNAs is variable, the most abundant being the transcript coding for N protein [18]. Consequently, analysis of ribosome density on subgenomic RNAs confirmed that protein N is the most abundantly produced protein, followed by protein M [32]. In addition, ribosome profiling allowed the identification of translation initiation sites. In addition to all the predicted translation initiation sites, a number of unidentified ORFs and uORFs were detected. Intriguingly, a collection of reads, supposedly corresponding to initiating ribosomes, has been located on a CUG codon at position 59 that is located between SL2 and SL3 in the 5′UTR without any explanation so far [32].

**Fig. 2:**
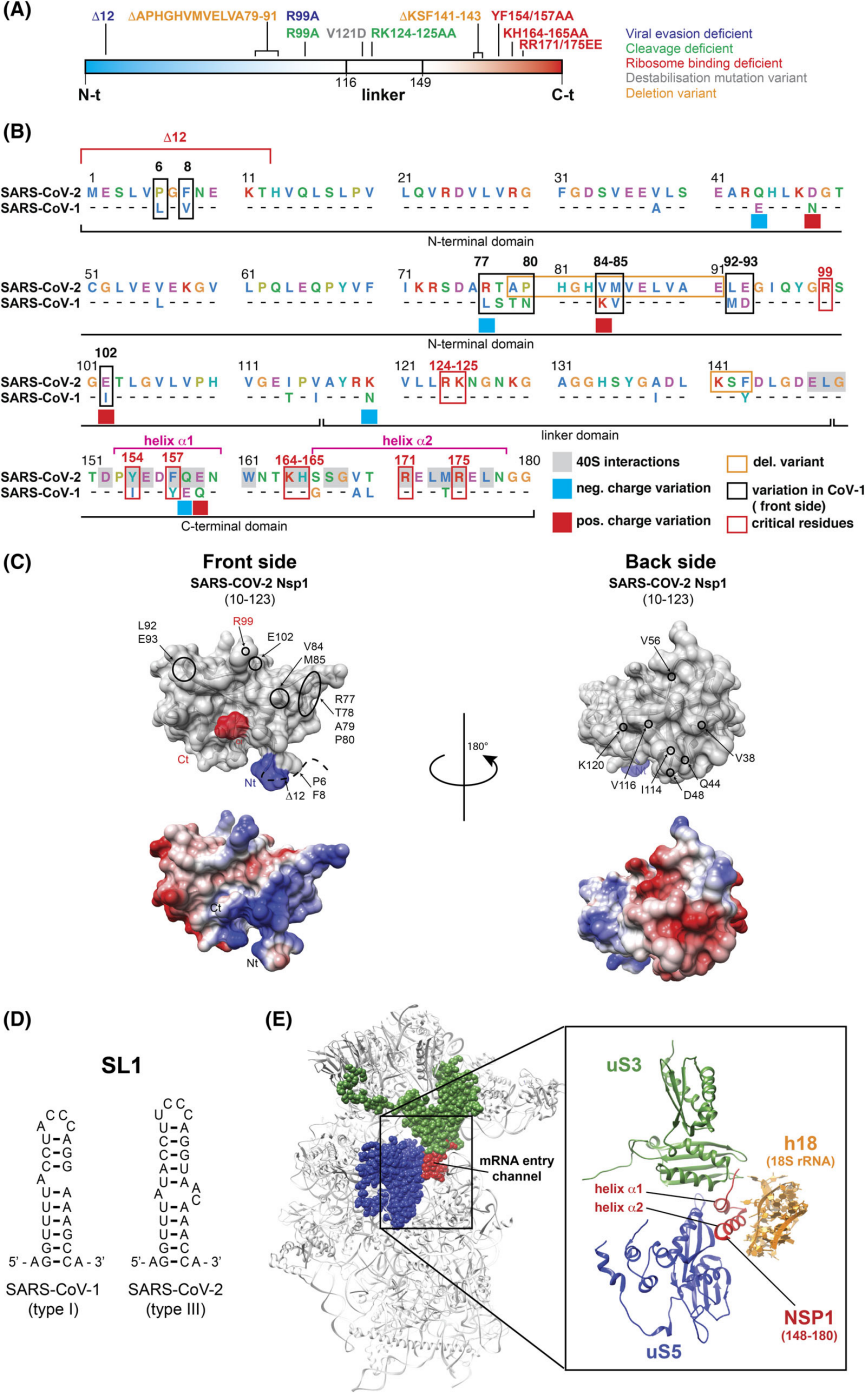
Non‐Structural Protein 1 or NSP1. (A) Linear representation of the three domains of SARS‐CoV‐2 NSP1: the Nt‐domain in blue, the linker domain and the Ct‐domain in red. The mutations of the residues that have been shown to be important for the functions of NSP1 are shown according to the color code indicated on the right. (B) Protein sequence alignment of SARS‐CoV‐2 and SARS‐CoV‐1 NSP1 proteins. For SARS‐CoV‐1, only the divergent amino acids are shown. The NSP1 proteins are subdivided into three domains: the N‐terminal domain, the central linker domain, and a C‐terminal domain. The amino acids are shown according to the following color code: negatively charged amino acids in pink, hydrophobic amino acids in blue, positively charged amino acids in green, aromatic amino acids in cyan, glycines and prolines in orange. Residues involved in interactions with ribosomal components are shaded in gray in SARS‐CoV‐2 [41, 42, 53]. Negative charge variations from SARS‐CoV‐2 to SARS‐CoV‐1 are indicated by blue squares, and positive charge variations are indicated by red squares. Residues that are divergent on the front side of NSP1 are boxed in black. Critical residues implicated in various functions of NSP1 are boxed in red. Deletions that have been found in SARS‐CoV‐2 variants are boxed in orange. (C) Surface representation of crystal structure of SARS‐CoV‐2 NSP1 from residues E10 to L123 (PDB: 7K7P) [50]. The upper panels are two views from the front (left) and back sides (right) of NSP1. The N‐terminal end is shown in in blue and the C‐terminal end in red. The position of residue R99 in SARS‐CoV‐2 NSP1 is indicated in red. Divergent residues from SARS‐CoV‐1 are circled in black. The lower panels represent the electrostatic surfaces of the protein with negative and positive charges colored in red and blue, respectively. (D) Secondary structures of SL1 from SARS‐CoV‐1 (left) and SARS‐CoV‐2 (right). (E) Cryo‐EM structure of the SARS‐CoV‐2 NSP1‐ribosomal 40S complex (PDB: 6ZLW) [41]. The C‐terminal domain of NSP1 is shown in red at the mRNA entry channel. The interactions between NSP1 (red) and the ribosomal proteins uS3 (green), uS5 (dark blue) and helix h18 of the 18S rRNA (orange) are shown.

During the early phase of infection, the genomic RNA is translated to produce polyproteins from ORF1a and ORF1ab, which are then further processed by proteolytic cleavages. The resulting non‐structural proteins (NSP1 to NSP16) are then produced in the cytoplasm of the infected cell and are among the first viral proteins to be expressed after virus entry. NSP1 is the first mature protein processed from polyproteins pp1a and pp1ab and is cleaved quickly following translation of the papain‐like protease activity (PL1^pro^) within NSP3. The start codon is embedded in SL5. After proteolytic processing, NSP1 consists of a 180‐amino acid protein that contains three domains: the N‐terminal domain, a linker domain, and a C‐terminal domain (Fig. 2A). NSP1 proteins are conserved in alpha‐ and betacoronaviruses, and therefore were being studied prior to the appearance of SARS‐CoV‐2 [34]. Early studies in SARS‐CoV‐1 have shown that NSP1 is responsible for efficient shut down of host cell translation [35, 36, 37]. Although the molecular mechanism was still unknown, a direct interaction between NSP1 and the host ribosome was discovered [38, 39]. In addition, it was found that NSP1 can recruit an uncharacterized nuclease that cleaves the host cellular mRNA in a co‐translational manner [40]. These pioneer studies enabled characterization of critical residues in NSP1 that are conserved in SARS‐CoV‐2 NSP1 (Fig. 2A). Indeed, mutations of residues KH164‐165 to alanines abolish the ability to bind to the 40S ribosomal subunit [38]. Mutations of residues RK124‐125 in the linker domain to alanines impair the cleavage guided by NSP1 [39]. These characterized mutations in SARS‐CoV‐1 turned out to be very useful information for structural and functional investigations of SARS‐CoV‐2 NSP1 and the mutations KH164‐165 also abolished binding to the 40S ribosomal subunit [41]. The structural data were also confirmed by mutations in α1 and α2 helices such as Y154A/F157A and R171E/R175E that also abolished ribosome binding [42]. Interestingly, viral transcripts are resistant to both NSP1 translation inhibition and NSP1‐guided RNA degradation. This phenomenon, called NSP1‐evasion, is mediated by a *cis*‐acting element: the hairpin SL1 that is present in all the viral transcripts [43, 44]. Although the molecular rationale of NSP1 evasion mediated by SL1 is not yet elucidated, it is clear that the N‐terminal domain of NSP1 is critical. Indeed, a small deletion of 12 amino acids is sufficient to destroy NSP1 evasion [45]. Similarly, mutation R99A, also located in the N‐terminal domain, abolishes not only NSP1 evasion but also NSP1‐guided cleavage [44]. During the Covid‐19 pandemic, many variants emerged; the coding sequence of NSP1 is a highly conserved region of the SARS‐CoV‐2 genome, but a few variants contained interesting mutations in NSP1. In the N‐terminal domain, an in‐frame deletion Δ500–532, which results in the deletion of residues A79 to E91, modifies the interferon I response by the host cell [46]. In the linker domain, the mutation V121D was found in the variant NIB‐1; although the real impact of this mutation was not investigated, it affects a highly conserved residue and its mutation is expected to destabilize NSP1 [47]. In addition, another deletion of three amino acids in the coding region of NSP1 was found in SARS‐CoV‐2 variants that were present in several countries. As the deleted residues KSF241‐143 are located in the C‐terminal domain, structural modeling studies suggested that the deletion decreased NSP1 ribosome binding [48]. Sequence comparison of NSP1 protein from SARS‐CoV‐1 and SARS‐CoV‐2, two members of the Sarbecovirus subgenus, revealed that NSP1 is highly conserved (Fig. 2B). The overall similarity is very high (91%), and the critical residues previously described are conserved between SARS‐CoV‐1 and SARS‐CoV‐2. However, several other key residues are variable in the three domains of NSP1. Structural studies by NMR and X‐ray crystallography enabled the elucidation of the three dimensional structures of the N‐terminal domain of SARS‐CoV‐1 [49] and SARS‐CoV‐2 NSP1 [50, 51]. In SARS‐CoV‐2 NSP1, the front side of the protein harbors a cluster of positively charged amino acids, whereas the back side is globally negatively charged (Fig. 2C). In addition, the residue R99, that is critical for NSP1 evasion, is located on the front side. As NSP1 evasion is mediated by the hairpin SL1 located in the 5′ leader of the genomic and subgenomic RNAs, the positively charged front side of NSP1 is more susceptible to interact with negatively charged nucleic acids. Concerning SL1 hairpins, slight but significant differences between the two viruses are found; indeed, SARS‐CoV‐1 contains type I SL1 in its 5′ leader, while SARS‐CoV‐2 has a type III SL1 [34] (Fig. 2D). Swapping experiments of key residues in both NSP1 and SL1 from SARS‐CoV‐1 and ‐2 have demonstrated that these two elements have actually co‐evolved thereby confirming the tight functional link between the NSP1 protein and SL1 hairpin [34]. In these structures of NSP1, the sole N‐terminal domain is visible because the remaining parts of NSP1 are intrinsically disordered [52]. Interestingly, when NSP1 is bound to the 40S ribosomal subunit, this feature is inverted, meaning that the C‐terminal domain becomes structured, whereas the N‐terminal domain becomes flexible. Consequently, the sole C‐t domain is visible at atomic resolution by cryo‐EM of the 40S‐NSP1 complex [41, 42, 53]. The binding of NSP1 to the 40S ribosomal subunit induces the folding of the C‐terminal domain into two helices α1 and α2 (Fig. 2E). The binding takes place at the mRNA entry channel through tight interactions between the two alpha helices and ribosomal proteins uS3 and uS5, and helix h18 of the 18S rRNA. An additional globular density, seemingly corresponding to the N‐terminal domain of NSP1, has been observed in the proximity of eS10, between uS3 and helix h16 of the rRNA. Indeed, its size is compatible with the estimated size of the N‐terminal domain of SARS‐CoV‐2 NSP1 [50, 51]. The position of the C‐terminal domain of NSP1 is incompatible with the presence of an mRNA in the mRNA channel, and therefore, translation is impossible when NSP1 is bound to the 40S ribosomal subunit because of steric hindrance to the access of the mRNA channel. NSP1 acts like a genuine plug in the mRNA channel. NSP1 does not prevent mRNA binding to the ribosome, as a ribosomal 40S complex programmed with CrPV IRES RNA has been observed by cryo‐EM [53]. However, the IRES is not properly accommodated in the mRNA channel, suggesting that NSP1 interferes with this critical step. Moreover, NSP1 locks the head to the body of the 40S ribosomal subunit and maintains a so‐called closed‐state conformation that prohibits mRNA loading into the channel [53]. The binding site of NSP1 on the 40S ribosome overlaps with the binding site of eIF3j, a critical translation initiation factor that is essential for mRNA loading in the mRNA channel. Single molecule approaches have demonstrated that NSP1 is actually competing with eIF3j and thereby inhibits pre‐initiation complex formation [54]. Another translation factor, eIF1, which binds on the other side of the mRNA channel, induces conformational changes that allosterically increase the affinity of NSP1 for its 40S binding site [54]. An important issue to better understand the role of NSP1 in the infectious program is to determine how and when NSP1 binds to the ribosome. Structural studies by cryo‐EM have led to structures of various complexes containing NSP1, such as 40S, 43S, and empty 80S (without mRNA), suggesting that NSP1 can enter into the ribosome at any stage of translation initiation or ribosome recycling [41]. Under normal physiological conditions, the ratio of empty 80S in the cell with an accessible mRNA channel has been estimated to be around 50% of the ribosome pool [55]. During viral infection, it is possible that the population of empty 80S is progressively increasing because cellular translation is gradually inhibited, thereby liberating ribosomes for viral translation. Interestingly, it has been shown that NSP1 is able to bind translating ribosomes in polysomes and stimulate translation termination [56]. Therefore, the first role of NSP1 is possibly to gradually hijack the ribosome pool for exclusive viral translation by forcing the termination of ongoing translating ribosomes. The second function would be to prevent *de novo* translation initiation of cellular mRNAs. Third, NSP1 bound to the ribosome allows specific translation of viral transcripts. During viral translation, so‐called NSP1‐evasion is guided by the *cis*‐acting SL1 hairpin, a structural element that is present in both genomic and subgenomic RNAs [43, 44, 45]. The fate of NSP1 during viral translation is not yet fully understood; according to one model, NSP1 remains attached to the ribosome during viral translation [43], while another model proposes that NSP1 is removed from the ribosome after viral mRNA accommodation in the mRNA channel [45]. Although the molecular mechanism is still not elucidated, NSP1‐evasion requires an intact N‐terminal domain, indicating that the signal of the presence of SL1 in the translated RNA might transit from the Nt‐ domain of NSP1 to the C‐t domain that is located in the mRNA channel. The need for another *trans*‐acting factor making intermediate contacts between SL1 and NSP1 cannot be excluded at this point. The probable allosteric mechanism that leads to the removal of the C‐terminal domain of NSP1 out of the mRNA channel remains to be characterized.

## Cellular translation

After virus entry, the first translation rounds of genomic RNA lead to the synthesis of NSP1 that will bind to ribosomes and stimulate translation termination of cellular mRNAs that are engaged in polysomes [56]. Then, the NSP1 ribosome plug seemingly blocks *de novo* translation of cellular mRNAs. However, the blockage is not complete, and subsets of cellular mRNAs are differentially impacted by NSP1‐mediated translation inhibition. Among these, subfamilies of cellular mRNAs escape this general inhibition and continue to be translated despite the presence of NSP1 on the host ribosomes. Indeed, ribosome profiling data indicate specific mRNA subclasses escape this translation inhibition. For instance, mRNAs encoding specific RNA Binding Proteins are still translated efficiently in the presence of NSP1 [57]. Similarly, TOP (5′ terminal oligo‐pyrimidine) mRNAs are also preferentially translated in the context of NSP1 expression [57]. These mRNAs encode components from the translational machinery, such as ribosomal proteins and translation factors. In addition, Larp1, which is a key factor in the specific translation of TOP mRNAs, is required for their specific translation in the presence of NSP1 [57]. The rationale of this phenomenon is not yet fully understood, but it is tempting to propose that the viral strategy behind this point is that the virus needs an intact and functional host translational machinery, and therefore, TOP mRNAs need to be translated efficiently during the whole infectious program to maintain efficient viral translation. Although the molecular mechanism is still unknown, it will be interesting to investigate the putative *cis*‐acting elements that might be present in the TOP mRNA 5′UTRs, and the putative *trans*‐acting factors that are required to promote NSP1‐evasion.

**Fig. 3:**
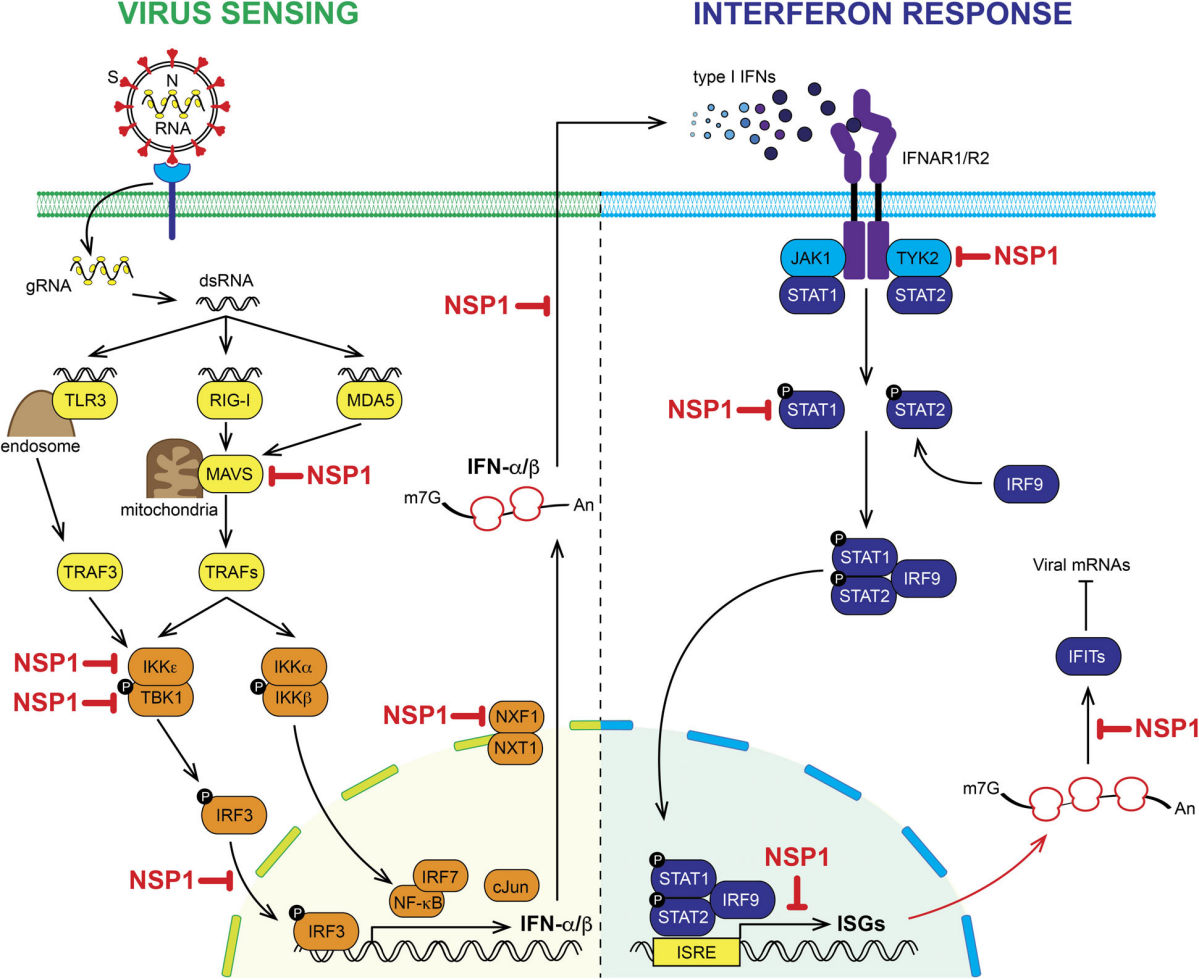
SARS‐CoV‐2 NSP1 interferes with the host antiviral responses. (Left) The entry of SARS‐CoV‐2 in the cytoplasm of the infected cell introduces double‐stranded RNA (dsRNA) that triggers the activation of innate immune pathways that lead to the production of type I interferon IFN‐α and ‐β which are secreted by the infected cell. (Right) The produced type I interferons will then activate, through a cascade of phosphorylations, the antiviral response by stimulating the expression of IFN‐Stimulated Genes (ISGs). The SARS‐CoV‐2 viral protein NSP1 shuts down the antiviral response by interfering with multiple steps of this pathway (the steps inhibited by NSP1 are shown in red).

In contrast to the TOP mRNAs that are resistant to NSP1, other mRNA subclasses are hypersensitive to NSP1‐mediated translational inhibition. Among these, mRNAs that encode proteins involved in the host innate immune response are primarily inhibited by NSP1. Indeed, SARS‐CoV‐2 NSP1 prioritizes interference of multiple steps of the immune response pathway (Fig. 3) [41, 53]. Interferon alpha (IFN‐α) and beta (IFN‐β) are key players of the type I interferon response [58]. The signature of a viral infection by RNA viruses is the presence of double‐stranded RNA (dsRNA) in the infected cell (Fig. 3). The dsRNA molecules are sensed by three distinct receptors: the Retinoic‐acid Inducible Gene I (RIG‐I), the Melanoma Differentiated Associated‐5 (MDA5) (that are both located in the cytoplasm), and Toll‐Like Receptor 3 (TLR3) (which is present in the endosomal compartment) [59, 60]. In the cytoplasm, RIG‐I and MDA5 are activated upon RNA recognition and induce signaling cascades through the adaptor molecule Mitochondria Antiviral Signaling protein (MAVS), which is attached to the mitochondrial membrane [61]. Another pathway occurs through the endosome with the TLR3 sensor. Both pathways lead to the activation of TNF Receptor‐Associated Factors or TRAFs, which ultimately induce the phosphorylation of IRF3 and IKKβ [62]. Both factors are transcription activators of the two interferon‐α and ‐β subfamily genes (Fig. 3) [61]. After transcription, mature IFN‐α and IFN‐β mRNAs are exported to the cytoplasm for their translation. The produced interferon I proteins are then secreted and further bind to specific IFN membrane receptors (IFNAR1 and IFNAR2). Binding of type I IFNs to their cell surface receptors activate Janus Kinase I (JAK1) and Tyrosine Kinase 2 (TYK2), which phosphorylate STAT1 and STAT2. In their phosphorylated forms, a tripartite complex STAT1‐STAT2‐IRF9 will assemble and translocate to the nucleus. This complex, also called IFN‐Stimulated Gene (ISG) factor 3, binds on the IFN‐I‐Stimulated Response Element (ISRE), which is located upstream of all the ISG genes (Fig. 3). This will activate the expression of hundreds of ISGs that are required for an efficient antiviral response [63]. Coronaviruses are known to promote active repression of the host antiviral response at the beginning of the infectious program [59, 64]. This is also the case during SARS‐CoV‐2 infection and NSP1 is directly involved in this repression by reducing interferon I production [65, 66]. For instance, NSP1 was shown to directly interfere in the dsRNA cascade signaling pathway at the levels of MAVS, IKKε and TBK1. Studies on SARS‐CoV‐1 have shown that NSP1 directly targets IRF3 phosphorylation and affects localization in the nucleus [35]. As NSP1 from SARS‐CoV‐1 and ‐2 are highly conserved (Fig. 2B), this is very likely true also for SARS‐CoV‐2 NSP1. Indeed, viral proteins NSP1 and NSP13 from SARS‐CoV‐2 inhibit interferon activation, although the direct effect of NSP1 on IRF3 nuclear translocation has not yet been established [67]. Moreover, NSP1 also efficiently inhibits STAT1 and STAT2 phosphorylation [65]. Expression or phosphorylation of JAK1 and or TYK2 are also modified by NSP1 [68]. In the nucleus, SARS‐CoV‐1 NSP1 represses the transcription of ISGs [69]. NSP1 also impacts general mRNA metabolism by interfering with the export of mRNA from the nucleus to the cytoplasm by targeting the protein NXF1 in the receptor heterodimer NXF1‐NXT1 at the nuclear pore complex [70]. NSP1 interferes with the interactions between NXF1 and mRNA export adaptors and thereby impairs NXF1 docking at the nuclear pore. The consequence of this is an accumulation of mRNAs that are retained in the nucleus during SARS‐CoV‐2 infection [70]. In agreement with this latter study, the export of interferon mRNAs is particularly inhibited during infection, although the direct implication of NSP1 in the specific retention of interferon mRNAs has not been established yet [71]. Finally, as NSP1 binds to the ribosome, the translation rates of both interferon mRNAs and ISG mRNA are decreased [41, 45, 53, 66]. Altogether, NSP1 is a key molecule that is required to promote efficient evasion of the cellular antiviral responses.

Beside translation inhibition, NSP1 also mediates the specific degradation of targeted mRNAs. NSP1‐mediated cleavage of mRNAs was first shown in SARS‐CoV‐1 [36, 37, 38, 40, 72]. The binding of NSP1 to the 40S ribosomal subunit is essential for mRNA cleavage. In addition, the characterization of a mutant NSP1 RK124‐125AA (Fig. 2A), which can still bind efficiently to the ribosome but is not able to promote mRNA cleavage, led to proposal of the following model. These reports suggest that NSP1, while sitting on the ribosome, recruits a host ribonuclease that cleaves the targeted mRNAs in a co‐translational manner. The ribonuclease has not yet been characterized, and the molecular mechanism remains elusive. Concerning SARS‐CoV‐2, global mRNA degradation has been observed during early stages of infection, even prior to the induction of IFN genes [71]. Moreover, SARS‐CoV‐2 infection triggers activation of RNase L, a cellular ribonuclease that promotes widespread decay of host mRNAs [73]. Although RNase L may be involved in global mRNA decay during SARS‐CoV‐2 infection, NSP1‐mediated cleavages seem to be RNase L‐independent, suggesting that another host ribonuclease, yet uncharacterized, is involved as well [71]. As observed in SARS‐CoV‐1, it was also confirmed that host mRNA cleavages occur only in the context of NSP1 ribosome binding [44]. These studies also led to the characterization of the R99A mutation of the N‐terminal domain of NSP1 (Fig. 2A), a mutant that retained its ribosome binding capacity but does not promote mRNA degradation [44]. These important data will be useful to identify the host ribonuclease that is presumably recruited on the ribosome by NSP1. In addition, SL1 in the 5′ leader of SARS‐CoV‐2 genomic RNA interacts with 2′‐5′‐Oligoadenylate synthetase 1 (OAS1), which is a key enzyme driving the innate immune response to viral infection [74]. This interaction prevents the function of OAS1, which triggers the RNase L pathway.

## NSP1 structure and functions in other coronaviruses

Coronaviruses are pathogens that are affecting more and more animal species. Their aerial transmission promotes their rapid dissemination within dense human populations and intensive animal farms. Hundreds of animal species are infected by coronaviruses leading to host‐specific adaptations and progressive sequence divergence. Below, we will briefly describe the coronavirus species that infect humans and the consequent economic impact that other coronaviruses have on animal farming. The four genera of coronaviruses share a relatively similar gene organization with few variations. We will describe some of these gene idiosyncrasies and focus on the different roles of the NSP1 protein in the inhibition of cellular translation and viral immunity.

### Progressive onset of a new group of pathogenic viruses for humans

There are four species of endemic human coronavirus (HCoV) currently recognized by the International Committee for the Taxonomy of Viruses, namely, HCoV‐OC43, ‐229E, ‐NL63, and ‐HKU1, and three epidemic CoVs, including SARS‐CoV‐1 and 2 and MERS‐CoV.

The first coronaviruses isolated from human sources were identified in the mid‐1960s. The first human coronavirus, HCoV‐229E, was identified in 1966. In the following year, another HCoV named HCoV‐OC43 emerged. These first viruses were associated with the common cold. In 2002, SARS‐CoV‐1 appeared in Guangdong province of China, and the next year, the virus spread to more than 25 countries and caused 774 deaths. In the same decade, two more HCoVs, NL63 and HKU1, appeared in the Netherlands (2004) and Hong Kong (2005), respectively. In 2012, the highly pathogenic MERS‐CoV emerged in the Middle East and caused a total of 881 deaths with a 34.4% fatality rate. In late 2019, the pandemic originating from SARS‐CoV‐2 started. It was quickly world‐distributed and has so far caused 5.8 M deaths (February 2022).

**Fig. 4:**
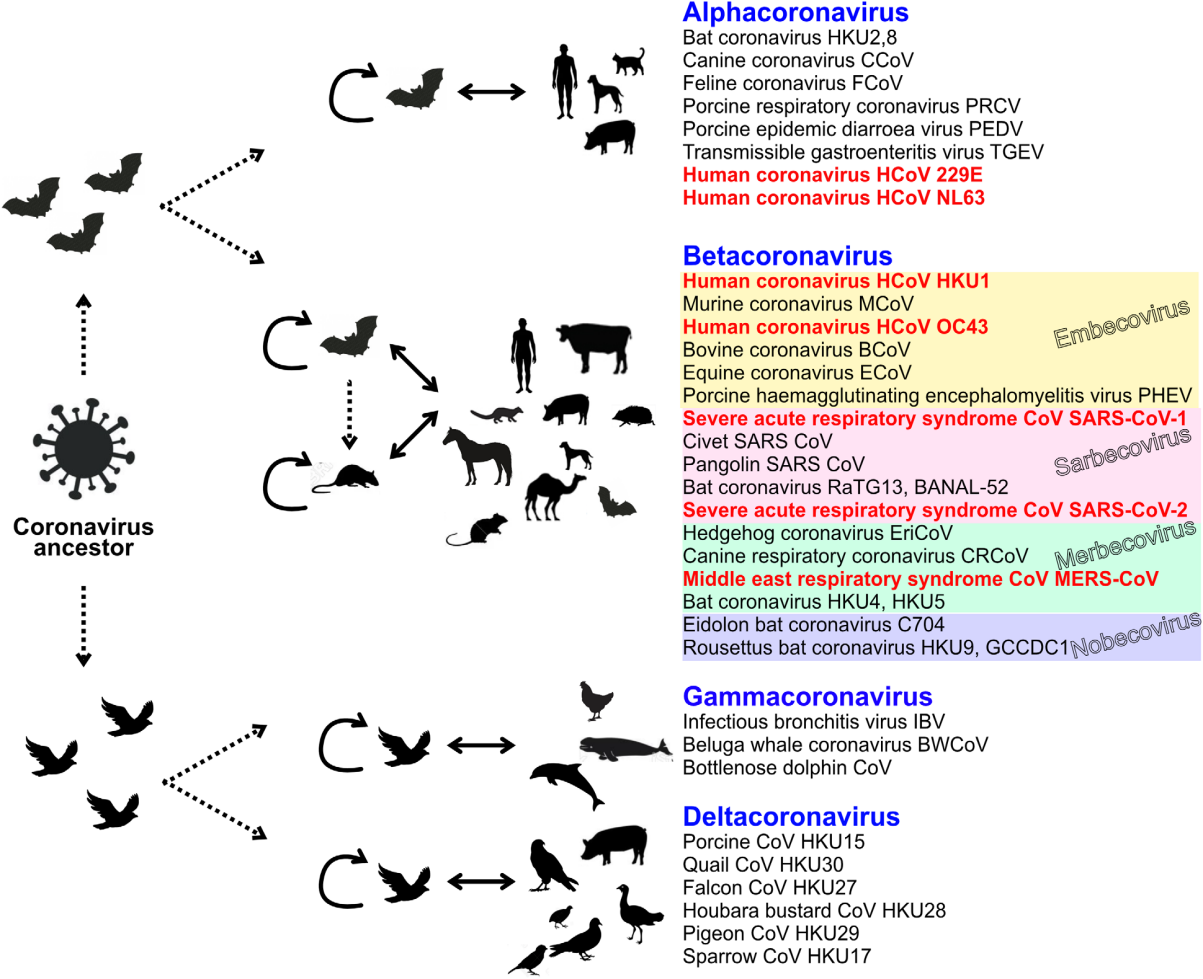
Classification of CoV genera and role of animals in transmission. The four genera *Alphacoronavirus, Betacoronavirus, Gammacoronavirus, and Deltacoronavirus* are shown. Some examples from each genus are given. The analysis includes the four main subgenera of *Betacoronavirus*: *Sarbecovirus*, *Embecovirus*, *Merbecovirus*, and *Nobecovirus*. The *Hibecovirus* subgenus containing only Bat Hp‐betacoronavirus Zhejiang 2013 is not shown. The diagram shows the possible role of animals in the transmission of the different coronaviruses, intermediate hosts, and potential ancestor origins.

Generally, human coronaviruses are believed to be a result of the zoonotic transfer or “spillover” from animal reservoirs, either directly or through an intermediate animal host [75]. Bats and birds are the main reservoirs of most coronaviruses, which are spilled over to humans through intermediate hosts such as civets (SARS‐CoV‐1), camels (MERS‐CoV), or rodents (HCoV‐OC43 and HCoV‐HKU1) (Fig. 4). For now, human coronaviruses are found in the Alpha‐ and Betacoronavirus genera, which have a similar genome length and structure. However, highly pathogenic SARS‐CoV‐1,2 and MERS‐CoV encode more accessory proteins and thus produce more sgRNAs than the lowly pathogenic hCoV‐OC43 and hCoV‐NL63 in infected cells, suggesting that these additional accessory proteins contribute to pathogenesis and severity of viral infections [76].

### Two alphacoronaviruses cause common colds

Human coronavirus 229E (HCoV‐229E) infects humans and bats. It enters its host cells, preferentially of the respiratory tract, by binding to the aminopeptidase N (APN). Along with the human coronavirus OC43 (HCoV‐OC43), it is one of the viruses responsible for the common cold. The species belongs to the genus Alphacoronavirus. Colds are mostly mild, but serious respiratory complications can occur in older or chronically ill people.

Human coronavirus NL63 (HCoV‐NL63) was identified at the end of 2004 in a seven‐month‐old child with bronchiolitis in the Netherlands [77]. Its host cell receptor is angiotensin‐converting enzyme 2 (ACE2). Infection with HCoV‐NL63 has been confirmed worldwide and is associated with many common symptoms and illnesses. The virus has a seasonal association in temperate climates and is found mainly in young children, the elderly and immune‐compromised patients. HCoV‐NL63 may be responsible for 5% of common respiratory illnesses.

**Fig. 5:**
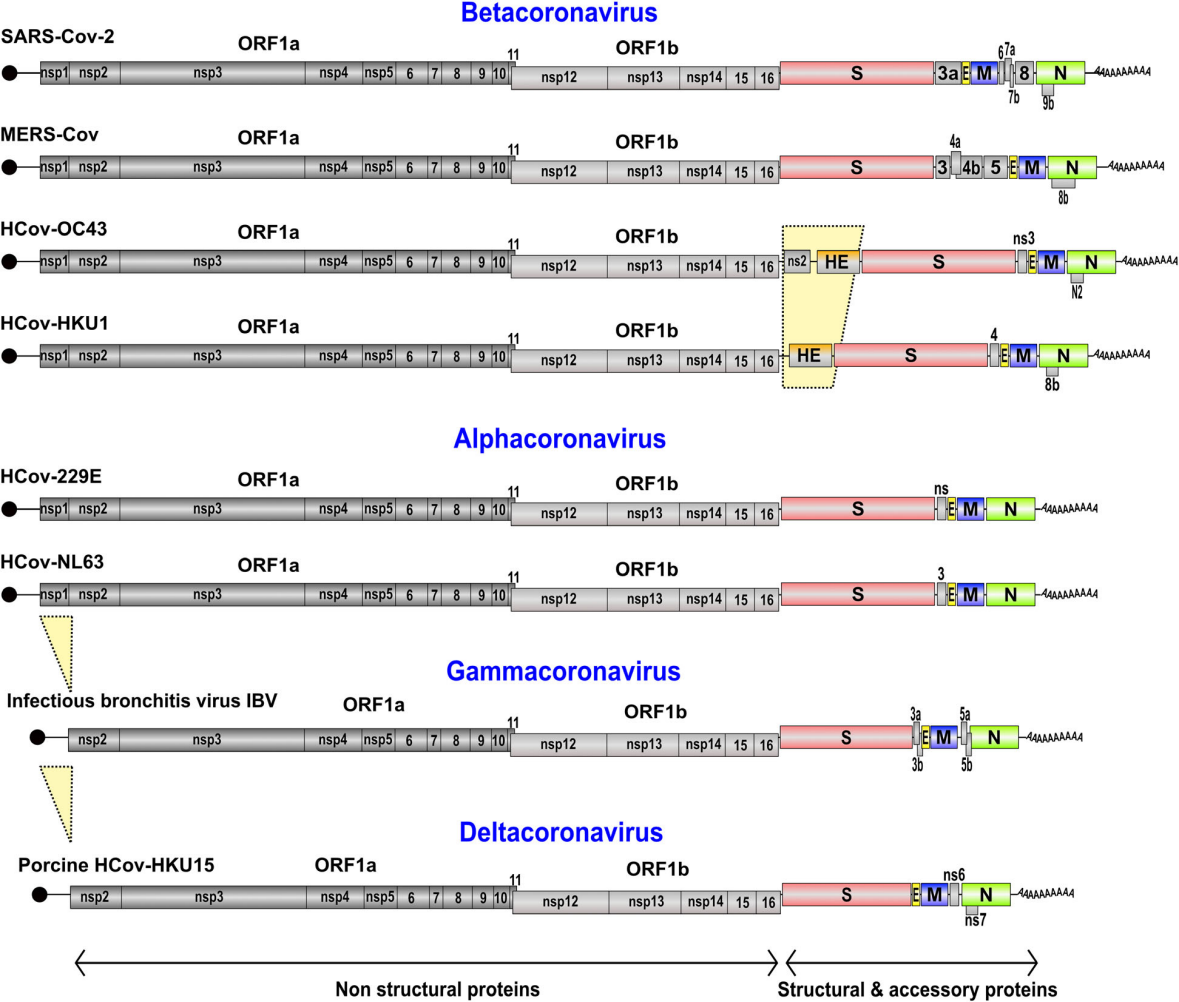
Genome organization of members in Beta‐, Alpha‐ and Deltacoronavirus genera. The genomic viral genomes are single‐stranded, positive‐sense RNA with a 5′ m^7^G‐cap (black circle) and a poly‐A tail (A_30‐60_) at the 3′ end. The genome encodes 16 non‐structural proteins (ORF1a: nsp1‐11 and ORF1b: nsp12‐16), 4 structural proteins (S, spike; E, envelope; M, membrane; N, nucleocapsid) and a varying number of accessory proteins (numbered boxes or ns). The upper 6 genomes are infectious for humans and are responsible for severe pathologies (SARS‐CoV‐2 and MERS‐CoV) or common pathologies (colds) (HCoV‐OC43, ‐HKU1, ‐229E, ‐NL63). HCoV‐OC43 and ‐HKU1 are characterized by a fifth structural protein: HE (hemagglutinin‐esterase). Infectious bronchitis virus (IBV) and Porcine CoV HKU15 are Gammacoronaviruses and Deltacoronavirus, respectively. They lack NSP1 protein and are among the smallest viruses in coronaviridae.

Both HCoV‐229E and HCoV‐63 have small genomes (about 27.3 kb) compared with other coronavirus and produce only one accessory protein and fewer sgRNAs than pathogenic SARS‐CoV and MERS‐CoV viruses (Fig. 5).

### Two betacoronaviruses of different animal origin cause common colds

The human coronavirus OC43 (HCoV‐OC43) is in the Embecovirus subgenus. It infects humans and cattle, and causes mild upper respiratory tract infections and only rarely severe pneumonia in neonates and aged people with underlying illnesses. The bovine coronavirus (BCoV) is the closest relative of HCoV‐OC43. It shares 97% nucleotide sequence identity across the entire genome length, (93.5% in the spike (S) gene, 98% in the envelope (E) gene). The recent ancestor of HCoV‐OC43 could be a coronavirus infecting cattle (BCoV), which would have adapted to humans during zoonosis. According to molecular clock studies, its emergence is relatively recent. With an estimated 4.39 x 10^−4^ substitutions per site per year, the time to the most recent common ancestor of HCoV‐OC43 and BCoV was dated to around 1890 [78]. HCoV‐OC43 has been proposed as a candidate for the 1889 to 1891 Russian flu pandemic which caused about one million deaths worldwide [79]. Together with human coronavirus HCoV‐229E, HCoV‐OC43 causes up to 30% of seasonal cold infections [80]. According to serological studies, infections with these two coronaviruses occur frequently in young children and then repeatedly throughout life [79].

Human coronavirus HKU1 (HCoV‐HKU1) is another Embecovirus of the genus Betacoronavirus [81]. Close to HCoV‐OC43 yet distinct, HCoV‐HKU1 arose from a different zoonotic progenitor and entered the human population independently. HCoV‐HKU1 originated from mice infected with murine CoV (MCoV). In humans, infection causes upper respiratory infections with cold‐like symptoms. It can progress to pneumonia and bronchiolitis. It was first discovered in January 2005 in patients in Hong Kong. Subsequent studies revealed that it had a worldwide distribution and a much earlier genesis.

Bats and birds are considered as the natural hosts for most of the HCoVs; however, HCoV‐OC43 and HCoV‐HKU1 evolved from a more distant ancestor that originated in mice [82]. Notably, HCoVs originating from mice express one more structural protein, hemagglutinin‐esterase (HE), in addition to the four major structural proteins (S, E, M, and N) (Fig. 5). HE proteins form homodimeric projections interspersed between the homotrimeric “peplomers” of spike protein. The HE lectin domain contributes to virion attachment and enhances sialate‐*O*‐acetyl‐esterase activity toward clustered sialo‐glycotopes [83]. However, the HE protein has lost the lectin function in HCoV‐OC43 and HKU1 as an adaptation to humans. The gene of HE was transmitted from influenza virus C/D to a proto‐Embecovirus via horizontal gene transfer [84].

Consequently, for entering the host cell, HCoV‐OC43 and HCoV‐HKU1 and other Embecoviruses originating from mice use 9‐*O*‐acetylated sialic acid as a viral receptor [85] in addition to a proteinaceous entry receptor via the spike protein. In murine CoV (MCoV or MHV), HE expression is dispensable for replication and rapidly lost during cell culture propagation. However, HE is critical for infection, and loss of HE‐associated acetyl‐esterase activity in HCoV‐OC43 abrogates the production of infectious virus [86]. It was also shown that acetyl‐esterase inhibitors dramatically reduce BCoV infectivity [87], and antibodies against HE neutralize the virus *in vitro* and *in vivo* [88].

The HCoV‐OC43 genome also contains a non‐structural protein gene (ns2) of 837 nucleotides downstream of ORF1ab (Fig. 5). Although not essential for viral growth, recent work has shown that the deletion of MCoV ns2 leads to a significant attenuation of the virus when inoculated into mice [89]. Protein ns2 contains a cyclic phosphate diesterase domain; it is also found in BCoV, Canine respiratory CoV (CRCoV), GiraffeCoV (GiCoV), etc. but not in HCoV‐HUK1.

### Gammacoronavirus and deltacoronavirus infections have huge economic impact on poultry and pig farming

Gammacoronaviruses cause avian infectious bronchitis in healthy galliform and non‐galliform birds. They are highly infectious and affect the respiratory, renal, and reproductive system. They cause significant decreases in weight gain and egg production in chickens and hens. Therefore, infections caused by Gammacoronaviruses induce significant economic losses in the poultry industry worldwide.

Chickens (*Gallus gallus*) are considered natural hosts of infectious bronchitis virus (IBV). These viruses have been reported to cause enteric diseases in turkeys, and renal and respiratory disease in pheasants. There is evidence regarding the identification of Gammacoronaviruses in healthy galliform and non‐galliform birds, suggesting the possibility that wild birds can carry IBV‐like viruses asymptomatically and scatter them widely. Gammacoronaviruses have also been identified in mammals, such as beluga whale, bottlenose dolphin, and Asian leopard cat; however, they primarily infect avian hosts [90, 91].

Deltacoronaviruses are the only coronavirus that can infect multiple species of mammals and birds. Avian Deltacoronavirus has been commonly reported in wild birds from different countries without any evidence of disease. Porcine Delta CoV (PDCoV) was initially identified in several avian and mammalian species, including pigs, in China in 2009‐2011. PDCoV has since spread worldwide and is associated with multiple outbreaks of diarrheal disease of variable severity in pig farms. PDCoV originated relatively recently from a host‐switching event between birds and mammals. So far, all other members of the Deltacoronavirus genus have been detected in birds, suggesting that birds are the natural host and ancestral reservoir of Deltacoronaviruses. PDCoV employs host aminopeptidase N (APN) as an entry receptor after interaction via spike (S) protein. PDCoV S protein targets the phylogenetically conserved catalytic domain of APN, which could explain its ability to infect many species. Binding of PDCoV to this interspecies‐conserved motif on APN could facilitate transmission to non‐reservoir species, including human and chicken. Interspecific contamination due to the remarkably broad reactivity with the APN cell receptor represents a significant epidemiological risk of poultry and pig farms [92, 93].

Gammacoronavirus genomes such as IBV typically contain ~27 700 bases. Deltacoronaviruses have the smallest known CoV genomes (25 400–26 700 bases). The genomic organization is similar to that of other CoVs, except that the NSP1 protein is not found in the gammacoronavirus or deltacoronavirus lineages, which code a distant homolog of SARS‐CoV NSP2 at the N‐terminus of polyprotein1a (Neuman et al. 2014 [94]) (Fig. 5). This main difference in the 5′ end of polyprotein 1a is often considered to be a genus‐specific marker. In the alpha and betacoronavirus genera, NSP1 proteins differ in size between ~110 and 245 amino acids [34, 95]. However, despite highly divergent sequences, NSP1 always exhibits similar functions to induce translational suppression and to evade host responses [35, 43, 96, 97]. The absence of NSP1 in the Gamma and Deltacoronaviridae raises the question of whether the lack of NSP1 in these virus families is compensated by another viral protein. Interestingly, the IBV Gammacoronavirus uses its accessory protein 5b to induce host protein synthesis shutoff. Therefore, orf5b is a functional equivalent of NSP1, although it is not produced at the initial stages of infection such as NSP1, but after later synthesis of subgenomic RNAs [98].

### NSP1 functional similarities and mechanistic divergences in alpha and betacoronaviruses

Despite sequence divergence across the Alpha and Betacoronavirus genera, the NSP1 protein uses a conserved two‐pronged strategy to suppress host protein translation, by inactivating the function of the 40S subunit and inducing host mRNA degradation. Although there is functional similarity, there is mechanistic divergence between SARS‐CoVs NSP1 and MERS‐CoV NSP1. First, the distribution of MERS‐CoV NSP1 in both the cytoplasm and the nucleus is in marked contrast to the localization of SARS‐CoV NSP1 exclusively in the cytoplasm. Second, MERS‐CoV NSP1 does not associate tightly with the 40S subunit, in contrast to SARS‐CoV NSP1. It results in a different strategy to inhibit host gene expression and facilitate the expression of MERS‐CoV‐infected cells. In the nucleus, MERS‐CoV NSP1 selectively targets cellular mRNAs by binding to mRNA‐binding proteins that form host mRNP complexes transported to the cytoplasm. Once transported into the cytoplasm, MERS‐CoV NSP1 inhibits translation and induces degradation of the nuclear‐encoded mRNAs, whereas MERS‐CoV mRNAs that are transcribed in the cytoplasm escape the inhibitory effects of NSP1 [99].

Similarly, NSP1 of transmissible gastroenteritis virus (TGEV), an Alphacoronavirus, is distributed in both the nucleus and the cytoplasm, and is unable to bind 40S ribosomal subunits. TGEV NSP1 shares with SARS‐CoVs NSP1 and MERS‐CoV NSP1 the common biological function of inhibiting host protein translation, but it lacks the activity to induce host mRNA degradation [100].

## Conclusion

During SARS‐CoV‐2 infection, NSP1 is required to complete the infectious program. NSP1 specifically targets the host ribosomes by acting like an mRNA channel plug to block host mRNA translation. NSP1 might be considered as a molecular lock that is opened specifically by viral transcripts that all contain the molecular key SL1. The tight functional link between NSP1 and SL1 is critical not only for efficient viral translation, but also to ensure complete host translation shut down. Host translation arrest has two main consequences: first, hijacking of translational machinery for viral translation, and second, blockage of host immune antiviral responses. This indicates that interfering with the tight interaction between NSP1 and the hairpin SL1 will not only have dramatic impacts on viral translation of genomic and subgenomic RNAs, but also enable efficient host immune responses. Consequently, NSP1 and SL1 are drug targets of choice for antiviral therapeutic strategies. Indeed, the first attempts using locked nucleic acid antisense oligonucleotides complementary to SL1 were shown to hinder viral replication *in vitro* and to protect transgenic mice from lethality when infected with SARS‐CoV‐2 [101].

## Conflict of interest

The authors declare that no competing interests exist.

## Author contributions

GE and FM generated the original figures and wrote the manuscript.
